# Expression of ETS1 in gastric epithelial cells positively regulate inflammatory response in *Helicobacter pylori-*associated gastritis

**DOI:** 10.1038/s41419-020-2705-8

**Published:** 2020-07-01

**Authors:** Yongsheng Teng, Baocheng Cang, Fangyuan Mao, Weisan Chen, Ping Cheng, Liusheng Peng, Ping Luo, Dongshui Lu, Nan You, Quanming Zou, Yuan Zhuang

**Affiliations:** 1 National Engineering Research Centre of Immunological Products, Department of Microbiology and Biochemical Pharmacy, College of Pharmacy and Laboratory Medicine, Third Military Medical University, Chongqing, China; 2 The 988 Hospital of PLA, Henan Zhengzhou, China; 3 La Trobe Institute of Molecular Science, La Trobe University, Bundoora, VIC 3086 Australia; 4 Department of Hepatobiliary Surgery, Xinqiao Hospital, Third Military Medical University, Chongqing, China

**Keywords:** Cell signalling, Helicobacter pylori

## Abstract

Gastric epithelial cells (GECs) provide the first point of contact of the host by *Helicobacter pylori* (*H. pylori*), and the interaction between *H. pylori* and GECs plays a critical role in *H. pylori*-associated diseases. Aberrant expression of transcription factors (TFs) contributes to the pathogenesis of inflammatory disorders, including *H. pylori*-associated gastritis. ETS (E26 transformation specific) transcription factor family is one of the largest families of evolutionarily conserved TFs, regulating critical functions during cell homeostasis. We screened ETS family gene expression in *H. pylori*-infected mouse and human GECs and found that ETS1 (ETS proto-oncogene 1, transcription factor) expression was highly affected by *H. pylori* infection. Then, we reported that ETS1 was induced in GECs by *H. pylori* via *cagA* activated NF-κB pathway. Notably, we demonstrated that proinflammatory cytokines IL-1β and TNFα have synergistic effects on ETS1 expression during *H. pylori* infection in an NF-κB-pathway-dependent manner. RNA-seq assay and Gene-ontology functional analysis revealed that ETS1 positively regulate inflammatory response during *H. pylori* infection. Increased ETS1 is also detected in the gastric mucosa of mice and patients with *H. pylori* infection. Collectively, these data showed that ETS1 may play an important role in the pathogenesis of *H. pylori*-associated gastritis.

## Introduction

*Helicobacter pylori* (*H. pylori*) infects ~4.4 billion individuals worldwide and is closely associated with chronic gastritis^1–3^. Chronic gastritis induced by *H. pylori* can progress to atrophic gastritis, intestinal metaplasia, dysplasia, and ultimately gastric cancer (GC)^3^. Gastric epithelial cells (GECs) provide the first point of contact of the host for *H. pylori* and the interaction between *H. pylori* and GECs plays a critical role in *H. pylori*-associated diseases^3,4^. Transcription factors (TFs) exquisitely regulate specific gene expression in a cell and play an important role in the development and function of multicellular organisms. Aberrant expression of TFs contributes to the pathogenesis of inflammatory disorders^5^. Previous research has demonstrated *H. pylori* can interfere with multiple TFs in GECs, such as STAT3, NF-κB, and β-catenin, to mediate inflammatory response^6–8^. Although an increasing number of TFs have been explored in the *H. pylori*-infected GECs, further exploration is also needed.

ETS1 (ETS proto-oncogene 1, transcription factor), a member of the ETS (E26 transformation specific) TF family, plays an important role in inflammatory disorders, such as allergic lung inflammation^9^, atopic dermatitis^10^, and systemic lupus erythematosus^11^. The present study focuses on ETS1 participate in inflammatory response through regulating the development and function of immune cells^12,13^. However, in addition to the immune cells, ETS1 can also be expressed or induced in multiple cell types, including endothelial cells, fibroblasts, and cancer cells^12,13^. In gastric mucosa, it has been reported that ETS1 isn’t expressed in the normal GECs; however, ETS1 is upregulated in GC cells, and associated with tumor invasion and metastasis^14–16^. However, what factors affect ETS1 expression in GECs is currently not known. As *H. pylori* is one of the strongest risk factors for GC^17,18^, it is therefore important to explore ETS1’s potential role during *H. pylori* infection and chronic gastritis.

Here, increased ETS1 is detected in the gastric mucosa of patients and mice with *H. pylori* infection, and it is induced in GECs by *H. pylori* via the cytotoxin-associated gene A (*cagA*)-activated NF-κB pathway. RNA-seq assay and Gene-ontology functional analysis reveal that ETS1 positively regulate inflammatory response during *H. pylori* infection. Herein, we report that ETS1 may execute proinflammatory response of GECs during *H. pylori* infection.

## Results

### *H. pylori* induced ETS1 expression in GECs

**Fig. 1:**
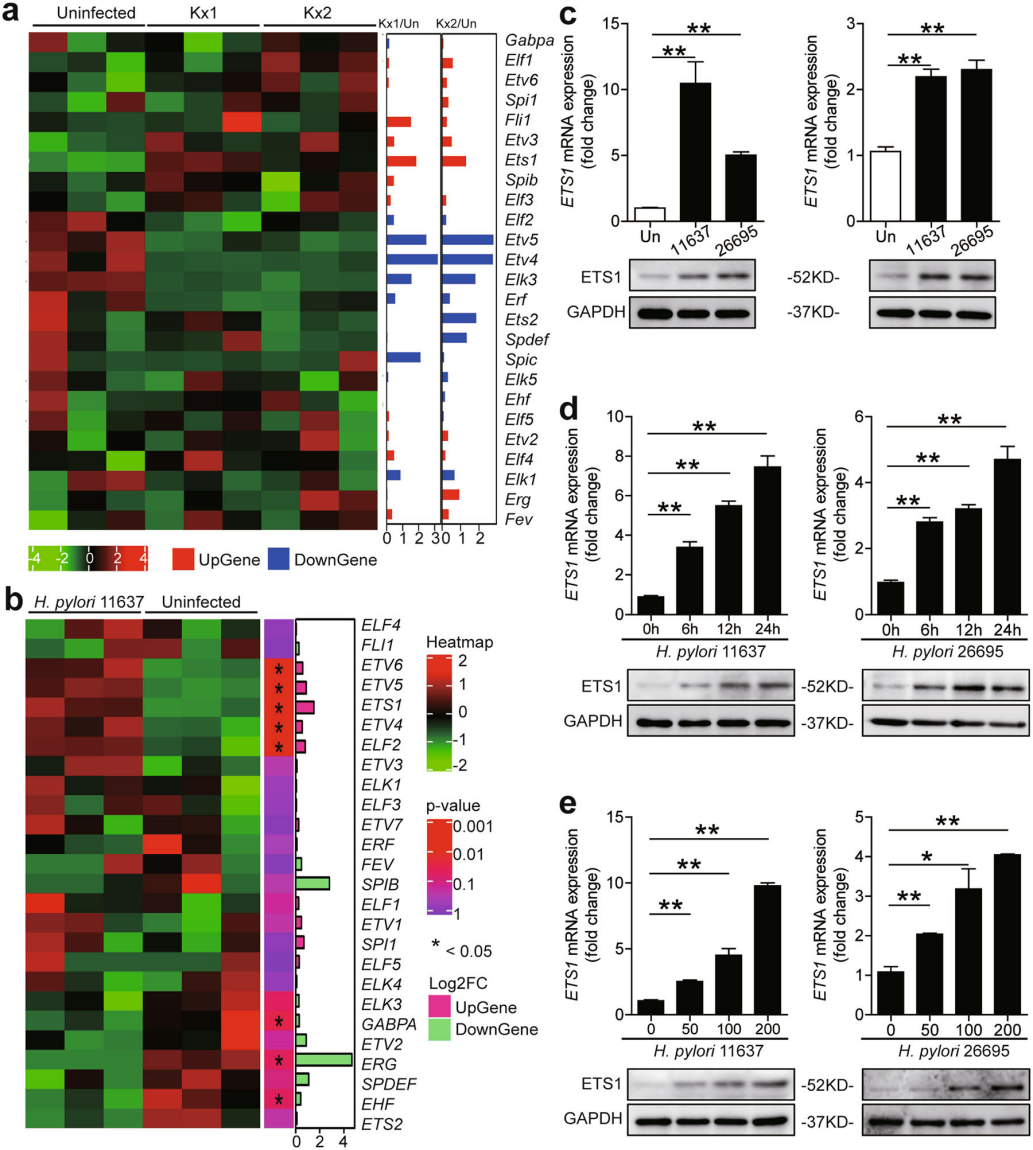
*H. pylori* induced ETS1 expression in GECs. **a** Relative expression of ETS transcription factor family genes in mouse gastric epithelial progenitor-derived cell line (mGEP) infected with clinically isolated *H. pylori* strains Kx1 and Kx2. The data were obtained from the GEO database (GSE10262). **b** RNA-seq analysis of expression of ETS transcription factor family genes in *H. pylori* 11637-infected AGS cells (*ETV3L* and *SPIC* were not detected). **c** ETS1 mRNA and protein expression in *H. pylori* 11637-, *H. pylori* 26695-infected and uninfected AGS cells (left) or human primary GECs (right) (MOI = 100, 24 h) were analyzed by real-time PCR (*n* = 3) and western blot. **d**, **e** ETS1 mRNA and protein expression in *H. pylori* 11637-infected or *H. pylori* 26695-infected AGS cells with different time points (MOI = 100) (**d**) or at different MOI (24 h) (**e**) were analyzed by real-time PCR (*n* = 3) and western blot. *P < 0.05, **P < 0.01.

Based on HUGO Gene Nomenclature Committee (HGNC) database, 28 human ETS family genes were found (Supplementary Table 1). To explore the relationship between *H. pylori* infection and ETS family gene expression in GECs, the relative expression of these genes in public microarray data from Gene Expression Omnibus (GEO) database containing expression profile data from mouse gastric epithelial progenitor-derived cell line (mGEP) infected by two *H. pylori* strains (chronic atrophic gastritis (ChAG)-associated Kx1 and gastric cancer-associated Kx2)^19^ were analyzed. We found that *Ets1* was the most increased ETS family genes induced by *H. pylori* (Fig. 1a). Curiously, we then screened the ETS family genes in *H. pylori* 11637-infected AGS cells by RNA-seq. Again, *ETS1* expression was the most increased (Fig. 1b). Similarly, AGS cells infected with *H. pylori* 11637 or 26695 showed increased ETS1 expression (Fig. 1c—left). Notably, we found that human primary GECs (EpCAM^+^) infected either *H. pylori* 11637 or 26695 also increased ETS1 expression (Fig. 1c—right). Furthermore, AGS cells infected with either *H. pylori* 11637 or 26695 increased ETS1 levels in an infection time- (Fig. 1d) and dose-dependent manner (Fig. 1e). Collectively, these findings clearly indicate that *H. pylori* infection induces ETS1 expression in GECs.

### *cagA* activated NF-κB pathway mediates ETS1 expression

**Fig. 2:**
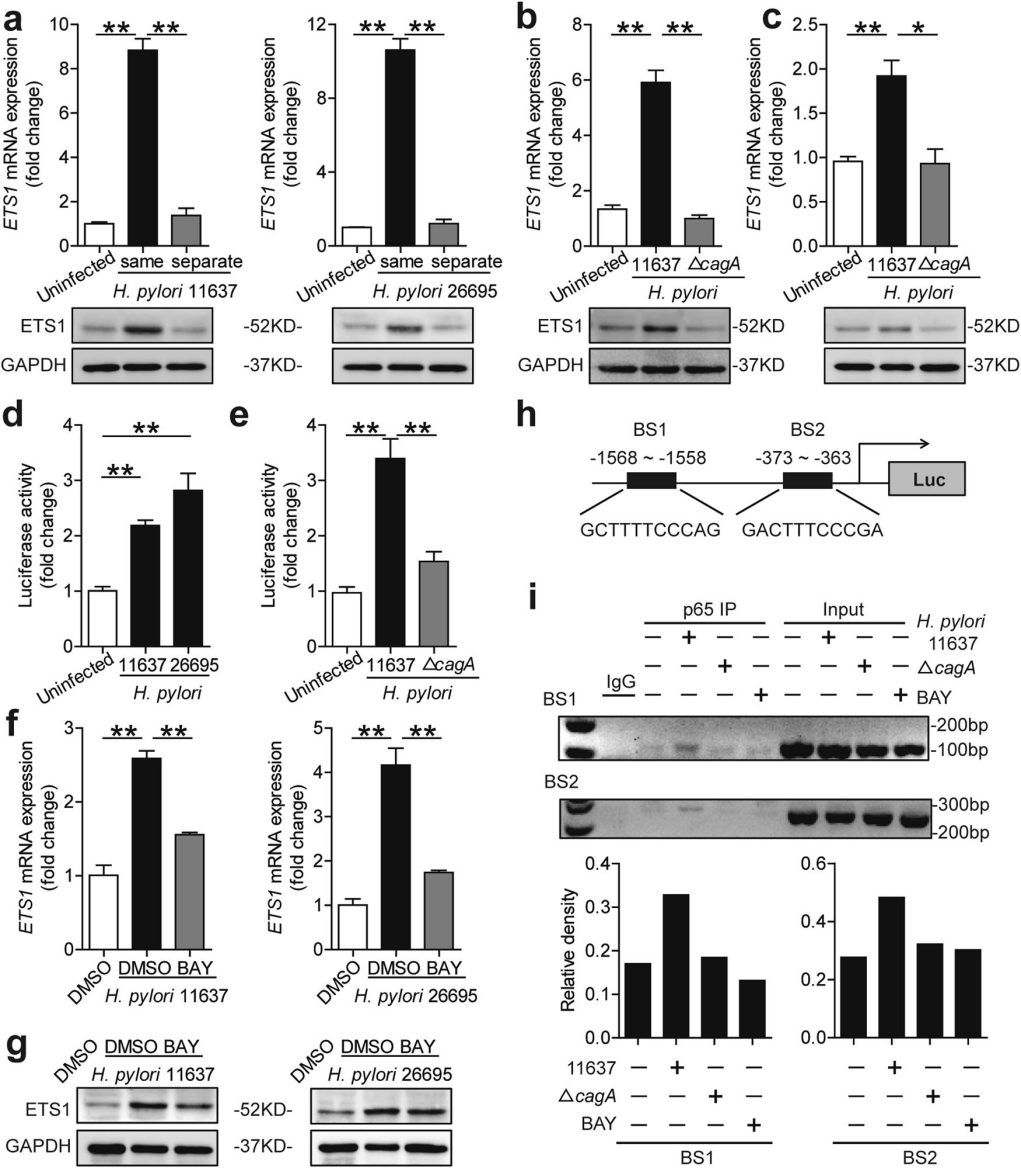
The *cagA* activated NF-κB pathway-mediated ETS1 expression. **a** AGS cells were either infected or not by *H. pylori* (MOI = 100) added in the same (lower) or separate (upper) chamber of a Transwell for 24 h. ETS1 mRNA and protein expression levels were analyzed by real-time PCR (*n* = 3) and western blot. **b**, **c** ETS1 mRNA and protein expression in *H. pylori* 11637- or Δ*cagA*-infected and uninfected AGS cells (**b**) or human primary GECs (**c**) (MOI = 100, 24 h) were analyzed by real-time PCR (*n* = 3) and western blot. **d** AGS cells were transfected with luciferase reporter constructs containing the *ETS1*-luc promoter for 4 h. Luciferase activity was measured to assess promoter activity after *H. pylori* 11637 or *H. pylori* 26695 infection (MOI = 100) for 24 h (*n* = 3). **e** AGS cells were transfected with luciferase reporter constructs containing the *ETS1*-luc promoter for 4 h. Luciferase activity was measured to assess promoter activity after *H. pylori* 11637 or Δ*cagA* infection (MOI = 100) for 24 h (*n* = 3). **f**, **g** AGS cells were pretreated with BAY 11-7082 (10 μM) and then infected with *H. pylori* 11637 or *H. pylori* 26695 (MOI = 100) for 24 h. ETS1 mRNA and protein expression were analyzed by real-time PCR (*n* = 3) and western blot. **h** The potential binding sites for p65 in the promoter of ETS1. **i** Chromatin immunoprecipitation (ChIP) assay in AGS cells infected with *H. pylori* 11637 (cells pretreated or not with BAY 11-7082 before infection) or Δ*cagA*, followed by regular PCR with primers designed for p65 binding site of ETS1 promoter region. The density of the bands was quantified and is shown on the below, and the column diagrams showed the “antibody group/input group” according to the results. * *P* < 0.05, **P < 0.01.

To determine whether *cagA* (a major virulence factor of *H. pylori*) contributes to ETS1 expression, we first carried out transwell infection assay. The results revealed that direct contact was required (Fig. 2a). As we all know, *cagA* can be injected into host cells via the type IV secretion system (T4SS). Notably, ETS1 levels only increased following infection with the *H. pylori* 11637 but not Δ*cagA* in AGS cells (Fig. 2b) or human primary GECs (Fig. 2c). To further confirm ETS1 is induced in *H. pylori*-infected GECs, we conducted the luciferase reporter assay using cells containing an *ETS1*-luc promoter. In contrast to the uninfected control, infection with *H. pylori* 11637 or 26695 significantly enhanced luciferase activity in AGS cells (Fig. 2d). Furthermore, enhanced luciferase activity was also *cagA* dependent (Fig. 2e). Next, we found that ETS1 expression in *H. pylori*-infected AGS cells was suppressed by inhibiting the NF-κB pathway using inhibitor BAY 11-7082 (Fig. 2f, g, SFig. 1a and b). Then, the PROMO tool V.8.3 of TRANSFAC^20,21^ (10 maximum matrix dissimilarity rate) showed that ETS1 promoter contains two p65 binding sites (binding site 1: GCTTTTCCCAG (−1568 to −1558); and binding site 2: GACTTTCCCGA (−373 to 363)) (Fig. 2h). Subsequently, a ChIP assay was performed. The results revealed that the *H. pylori* 11637-infected cells increased p65 binding to the ETS1 promoter; however, the Δ*cagA* did not, and BAY 11-7082 inhibited this binding (Fig. 2i). Collectively, these results showed that *cagA* activates the NF-κB pathway to mediate ETS1 expression.

### IL-1β and TNFα have synergistic effects on ETS1 expression during *H. pylori* infection

**Fig. 3:**
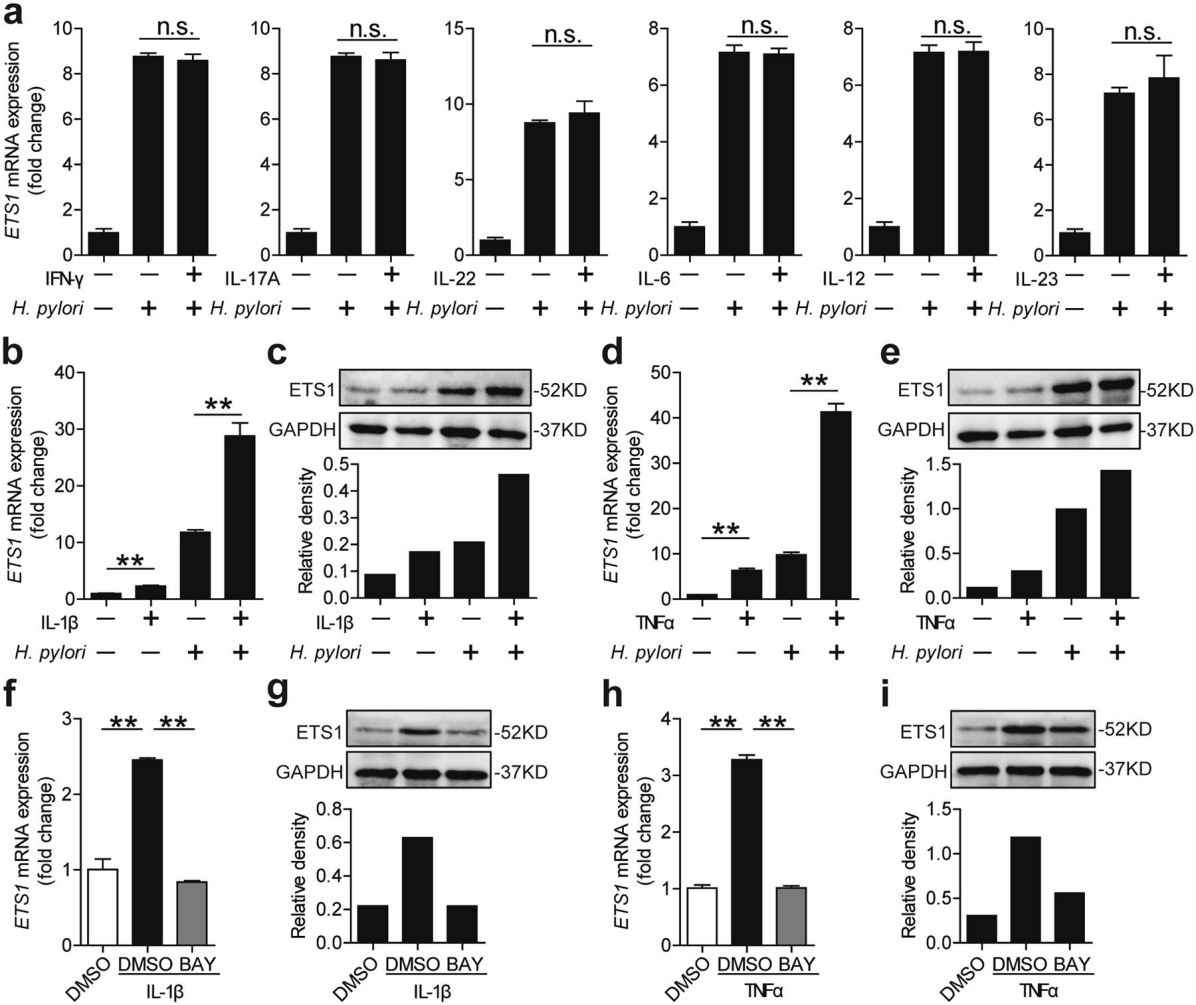
IL-1β and TNFα have synergistic effects on ETS1 expression during *H. pylori* infection. **a** ETS1 mRNA expression in AGS cells stimulated with *H. pylori* 11637(MOI = 100) and/or IFNγ, IL-17A, IL-22, IL-6, IL-12, or IL-23 (100 ng/ml) (24 h) were analyzed by real-time PCR (*n* = 3). The first three (IFNγ, IL-17A, and IL-22) are the results of the same batch, and the last three (IL-6, IL-12, and IL-23) are the results of another same batch. **b**, **d** ETS1 mRNA expression in AGS cells stimulated with *H. pylori* 11637(MOI = 100) and/or IL-1β (**b**) or TNFα (**d**) (100 ng/ml) (24 h) were analyzed by real-time PCR (*n* = 3). **c**, **e** ETS1 protein expression in AGS cells stimulated with *H. pylori* 11637(MOI = 100) and/or IL-1β (**c**) or TNFα (**e**) (100 ng/ml) (24 h) were analyzed by western blot. **f**, **g** AGS cells were pretreated with BAY 11-7082 (10 μM) and then treated with IL-1β for 24 h. ETS1 mRNA (**f**) and protein (**g**) expression were analyzed by real-time PCR (*n* = 3) and western blot. **h**, **i** AGS cells were pretreated with BAY 11-7082 (10 μM) and then treated with TNFα for 24 h. ETS1 mRNA (**h**) and protein (**i**) expression were analyzed by real-time PCR (*n* = 3) and western blot. Quantized expression of proteins were analyzed by Image Lab software (*n* = 1). n.s. *P* > 0.05, **P < 0.01.

Immune cell infiltration and inflammatory cytokine production are the characteristics of *H. pylori*-infected gastric mucosa^3^. Previous research has showed cytokines could modulate *H. pylori-*mediated gene expression in GECs, such as IFN-γ modulates *H. pylori*-mediated B7-H2 downregulation^22^, and IL-22 modulates *H. pylori*-mediated MMP10 upregulation in GECs^23^. Thus, we examined whether cytokines (IFN-γ, IL-17A, IL-22, IL-6, IL-12, IL-23, IL-1β, and TNFα) could modulate ETS1 expression in GECs. We showed that IFN-γ, IL-17A, IL-22, IL-6, IL-12, and IL-23 had no effect on modulating ETS1 expression during *H. pylori* infection (Fig. 3a). Notably, IL-1β and TNFα exerted a synergistic effect on *H. pylori*-mediated ETS1 expression (Fig. 3b–e). Then, the expression of ETS1 was suppressed by using BAY 11-7082 in IL-1β or TNFα stimulated AGS cells (Fig. 3f–i). Taken together, these findings suggest that IL-1β and TNFα have synergistic effects on *H. pylori*-mediated ETS1 expression through the NF-κB pathway.

### ETS1 positively regulate inflammatory response during *H. pylori* infection

**Fig. 4:**
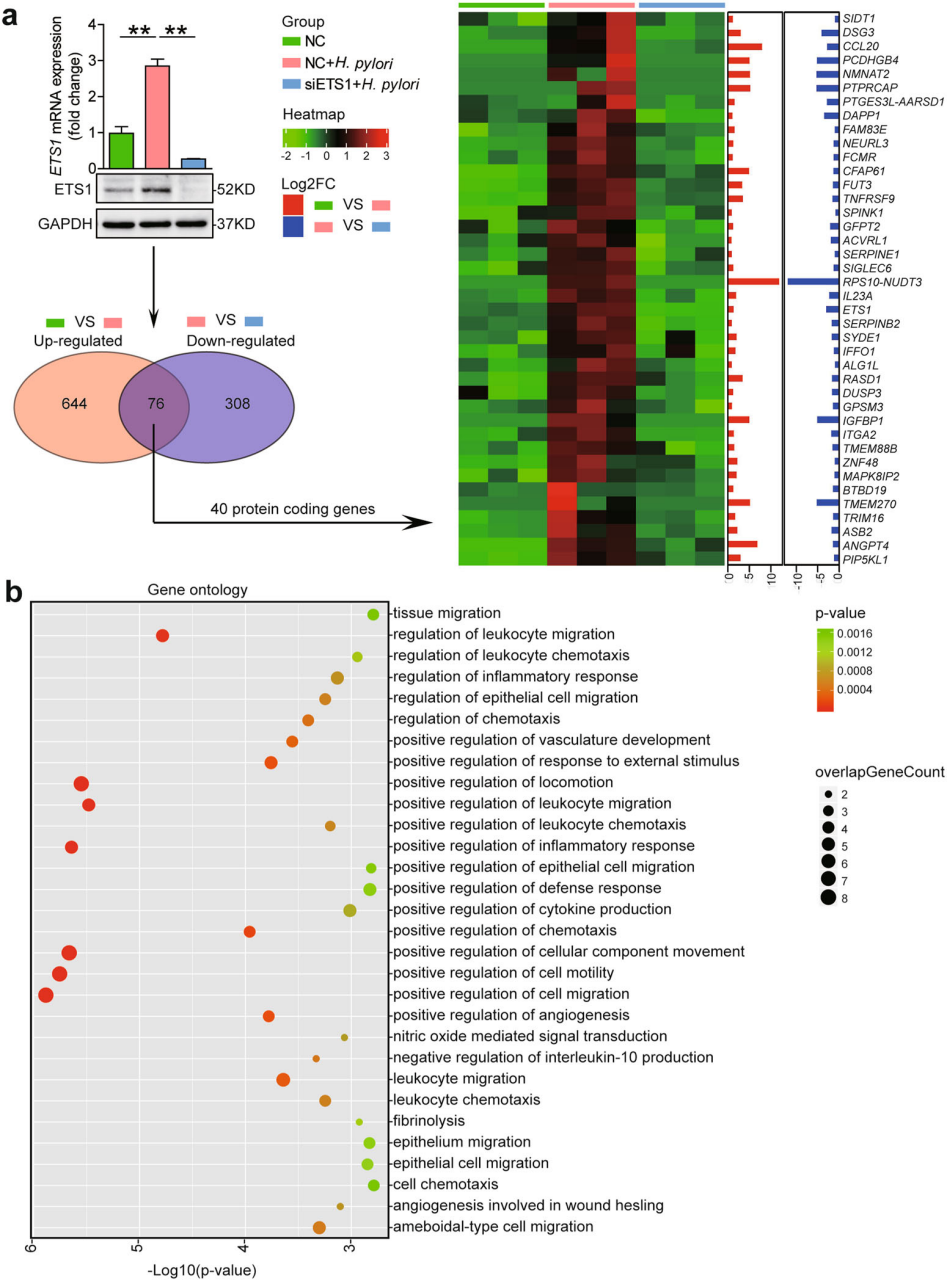
Analysis of ETS1 targets in GECs. **a** AGS cells transiently transfected with non-silencing siRNA (NC) or silencing siRNA directed to ETS1 (siETS1) for 24 h, and then infected with *H. pylori* 11637 (MOI = 100) for an additional 24 h. Subsequently, real-time PCR, western blot, and RNA-seq analysis were performed. RNA-seq analysis shows forty protein-coding genes were upregulated in *H. pylori* 11637-infected NC-transfected AGS cells and downregulated in *H. pylori* 11637-infected siETS1-transfected AGS cells. **b** Top thirty GO terms of Gene-ontology analysis of target genes. **P < 0.01.

To identify ETS1-positive regulate genes during *H. pylori* infection, we first suppressed ETS1 by siRNA followed by *H. pylori* infection, and then performed RNA-seq assay. RNA-seq analysis showed 76 genes were upregulated in *H. pylori* 11637-infected NC-transfected AGS cells and downregulated in *H. pylori* 11637-infected siETS1-transfected AGS cells (Fig. 4a). Among those genes, 40 protein-coding genes were founded (including ETS1) (Fig. 4a). Gene-ontology functional analysis reveals that the ETS1 is primarily involved in biological processes including positive regulation of cell migration, positive regulation of cell motility, positive regulation of cellular component movement, positive regulation of inflammatory response, positive regulation of leukocyte migration, and positive regulation of locomotion (Fig. 4b). Collectively, these results suggest that ETS1 positively regulates inflammatory response during *H. pylori* infection may through regulate leukocyte migration and chemotaxis.

### ETS1 is upregulated in mice and humans with *H. pylori* infection and its expression level is associated with the degree of gastric inflammation

**Fig. 5:**
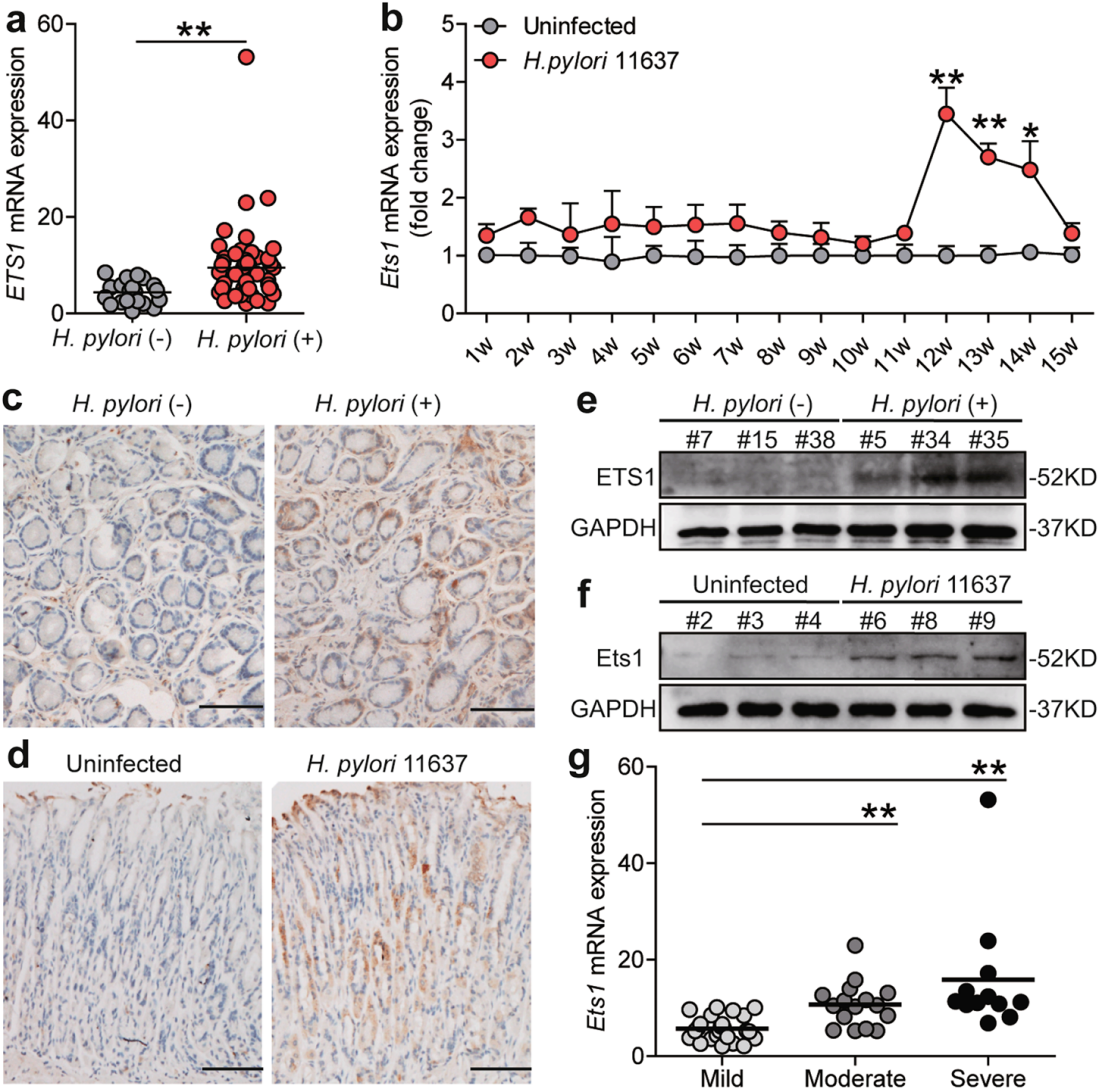
ETS1 is upregulated in mice and human with *H.pylori* infection and associate with the degree of gastric inflammation. **a** ETS1 mRNA expression in gastric mucosa of *H. pylori*-infected (*H. pylori* (+)) (*n* = 53) and uninfected donors (*H. pylori* (−)) (*n* = 20) were compared. **b** Dynamic changes of *Ets1* mRNA expression in *H. pylori* 11637-infected and uninfected wild-type C57BL/6 mice (*n* = 5). **c** ETS1 protein expression in gastric mucosa of *H. pylori* (+) and *H. pylori* (−) was analyzed by immunohistochemistry staining. Scale bar: 100 μm. **d** Ets1 protein expression in gastric mucosa of *H. pylori* 11637-infected and uninfected mice at 12 week p.i. was analyzed by immunohistochemical staining. Scale bar: 100 μm. **e**, **f** ETS1/Ets1 protein in human gastric mucosa of *H. pylori* (+) and *H. pylori* (−) or in mice gastric mucosa of *H. pylori* 11637-infected and uninfected mice at 12 week p.i. was analyzed by western blot. **g** ETS1 mRNA expression in gastric mucosa of *H. pylori* (+) patients with mild (*n* = 25), moderate (*n* = 16), severe (*n* = 12) inflammation were compared. *P < 0.05, **P < 0.01.

To determine whether *H. pylori* infection increases ETS1 expression in gastric tissues, we conducted ELISA (serology test for *H. pylori*-specific antibodies) and PCR (test for 16 S rDNA) assessments to distinguish between the *H.* pylori-infected and uninfected individuals. We found ETS1 expression increased in gastric tissues from *H. pylori*-infected individuals (Fig. 5a). Then, an animal model was established by infecting mice with *H. pylori* 11637, and dynamic changes of *Ets1* mRNA expression were detected. The results showed that *Ets1* mRNA expression reaching a peak of 12 weeks post-infection (Fig. 5b). Meanwhile, increased *Il-1β* and *tnfα* were also found in our mouse model (SFig. 2a, b). Furthermore, ETS1/Ets1 protein was indeed increased in the gastric mucosa of *H. pylori*-infected patients and *H. pylori*-infected mice, compared to those in uninfected patients or mice (Fig. 5e, f). Further, immunohistochemistry revealed that ETS1/Ets1 protein was increased in GECs with *H. pylori* infection (Fig. 5c, d). According to the *H. pylori*-infected gastric samples’ histopathological evaluation, the mild, moderate, and severe gastritis were divided. The levels of ETS1 expression was significantly correlated with the severity of gastritis (Fig. 5g). Collectively, these findings indicated that ETS1 is upregulated in mice and humans with *H. pylori* infection and may participate in the regulation of gastric inflammation.

## Discussion

GECs infection by *H. pylori* result in multiple TF activation and increased inflammatory cytokine production, changes in biological characteristics, and ultimately oncogenic transformation^6,24,25^. ETS family is one of the largest families of evolutionarily conserved TFs, regulating critical cellular functions during cell homeostasis and contributing to tumor progression when perturbed^26^. In the present study, we screened ETS family gene expression in *H. pylori*-infected mouse and human GECs, the results identified ETS1 as a key regulator. ETS1 was upregulated in mouse and human GECs with *H. pylori* infection, which was also confirmed by immunohistochemistry of biopsy specimens from patients. Notably, we also demonstrated that the ETS1 expression level in gastric mucosa was significantly correlated with the severity of gastric inflammation. Altogether, our findings demonstrate that ETS1 can be induced by *H. pylori* and ETS1 participates in proinflammatory response.

Previous studies have shown that the pathogenicity of *H. pylori* is largely attributed to virulence factor, *cagA* protein, which is a 120–140 kDa protein and can be injected into GECs via the T4SS^27^. *cagA* has an important role in the proinflammatory response of GECs during *H. pylori* infection^7,28^. Using a *cagA*-KO mutant strain, we showed that ETS1 expression is largely *cagA* induced. It has been well established that *cagA* injected into host cells perturbs multiple central signaling pathways, especially NF-κB signaling pathway^29–31^. The NF-κB signaling pathway is a master regulator of inflammatory responses as it regulated in many cellular processes, including proliferation, angiogenesis, and even transformation^32^. Then, our findings demonstrated that *cagA* activated transcription factor NF-κB-p65 directly upregulate ETS1 expression.

*H.* pylori-infected gastric mucosa produces many proinflammatory cytokines, and the resultant inflammatory microenvironment could, in turn, modulate *H. pylori*-mediated gene expression in GECs^22,23,33^. In the present study, we found that IL-1β and TNFα have synergistic effects on *H. pylori*-mediated ETS1 expression through the NF-κB signaling pathway. The results revealed that ETS1 plays a pivotal role in *H. pylori* infection. To analyze the ETS1-controlled gene program, an RNA-seq assay was performed, and Gene-ontology functional analysis reveals that ETS1 positively regulate the inflammatory response. And we also found that ETS1 positively regulate leukocyte migration and chemotaxis. Therefore, ETS1 positively regulates inflammatory response may through regulate leukocyte migration and chemotaxis. However, the precise molecular mechanisms that ETS1 regulate leukocyte migration and chemotaxis also need further exploration.

In conclusion, our findings demonstrate the vital role of inflammation and *H. pylori*-mediated activation of the NF-κB pathway in ETS1 regulation in gastric gastritis. Meanwhile, ETS1 has a proinflammatory role in gastric gastritis was founded. Notably, these findings reveal that ETS1 may play a crucial role in the pathogenesis of *H. pylori*-associated gastritis.

## Materials and methods

### *H. pylori* culture

The *cagA*-positive *H. pylori* NCTC 11637 (ATCC 43504, called *H. pylori* 11637 in this article), *cagA*-KO mutant *H. pylori* NCTC 11637 (called Δ*cagA* in this article), and *cagA*-positive *H. pylori* 26695 (ATCC 700392) were cultured on brain-heart infusion plates containing 10% rabbit blood at 37 °C under microaerophilic conditions.

### Cell culture and stimulation

The isolation of human primary GECs (EpCAM^+^) from tissues were performed as previously described^34^. Human primary GECs and AGS (ATCC) were cultured with DME/F12 (AGS) or RPMI 1640 (Human primary GECs) medium supplemented with 10% fetal bovine serum (FBS) at 37 °C in 5% CO_2_. Cells were stimulated with *H. pylori* 26695, *H. pylori* 11637, or Δ*cagA* at the indicated multiplicity of infection (MOI) and time. For *H. pylori* transwell infection^35^, cells were incubated in the presence of *H. pylori* that were added to the same (lower) or separate (upper) chamber of a 0.4 μm transwell inserts for 24 h. For cytokines stimulation, cells were stimulated with *H. pylori* 11637 (MOI = 100) and/or IFN-γ (100 ng/ml), IL-17A (100 ng/ml), IL-22 (100 ng/ml), IL-6 (100 ng/ml), IL-12 (100 ng/ml), IL-23 (100 ng/ml), IL-1β(100 ng/ml), or TNFα (100 ng/ml) for 24 h. For signal pathway inhibition experiments, cells were pretreated with BAY 11-7082 (10 μM) or DMSO for 2 h, then, infected with *H. pylori* 11637 or 26695 for 24 h. After co-culture, cells were collected for real-time PCR and western blot.

### Real-time PCR analysis

RNAiso Plus extracted total RNA from biopsy specimens, mouse gastric tissues and cultured cells were reverse-transcribed to cDNA by PrimeScript^TM^ RT reagent Kit. For mouse samples, expression levels were normalized to β-actin. And for human samples, GAPDH served as the normalizer. Real-time PCR was performed on the CFX96 (Bio-Rad, Hercules, USA) with the SYBO Green Real-time PCR Master Mix according to the manufacturer’s specifications. The relative gene expression was expressed as fold change calculated by the ΔΔCt method. The sequences of specific primers are presented in Supplementary Table 3.

### Western blot

In brief, equivalent amounts of cell or tissue lysates were resolved in 10% SDS-PAGE gels, and proteins were then transferred onto PVDF membranes and western blots were performed. 5% BSA was used for blocking the PVDF membranes. In turn, membranes were incubated with specific antibody (Ab). This was followed by incubation with HRP-conjugated secondary Abs. Detected proteins were visualized by using ECL chemiluminescence kit. Protein levels were quantified using the Image Lab software, version 3.0 (Bio-Rad, USA).

### Luciferase reporter assay

Promoter constructs containing the region from −2000 to 0 of the ETS1 gene were amplified from human genomic DNA by PCR (constructed by Sangon Biotech, Shanghai, China). The promoter sequences (-2000/0) (Supplementary Table 2) were obtained from The Eukaryotic Promoter Database (EPD, https://epd.epfl.ch//index.php). The amplified sequences were cloned into the NheI and HindIII sites of the pGL3-basic vector (Promega, Madison, USA). Cells were transfected with pGL3-*ETS1* and internal control pRL-TK (Promega, Madison, USA) by using Lipofectamine 2000 according to the manufacturer’s protocol for 4 h. Next, cells were infected with *H. pylori* 26695, 11637 or Δ*cagA* (MOI = 100). The luciferase activities in the cell lysates were measured using the Dual-Luciferase Reporter assay after 24 h following the manufacturer’s protocol.

### Chromatin immunoprecipitation (ChIP) assay

Cells were infected with *H. pylori* 11637 (cells pretreated with or without BAY 11-7082) or Δ*cagA* for 18 h. Then, the EZ-Magna ChIP™ A/G Chromatin Immunoprecipitation Kit was used following the manufacturer’s protocol. The sequences of primers for ETS1 promoter were as follows: BS1, forward, 5’-GTGGTTCATTTGGACGTGTAAATGT-3’; reverse, 5’-TGCAATCCAGCTGTGTTTCCA-3’ (product length 141 bp); BS2, forward, 5’-AGGACACGGGCTCACGAATC-3’; reverse, 5’-GGAGGAGCAGTGCGTGGAG-3’ (product length 293 bp). The band densities were quantified using the Image J software (NIH, Bethesda, MD, USA). The column diagrams showed the “antibody group/input group” according to the results.

### Small interfering RNA (siRNA) transfection

siRNA targeting *ETS1* and control siRNA were purchased from GenePharma (Shanghai, China). The ETS1-targeting siRNA (siETS1) sequences were: sense, 5’-GCAGCCAGUCAUCUUUCAATT-3’, and antisense, 5’-UUGAAAGAUGACUGGCUGCTT-3’. The control siRNA (Negative control (NC)) sequences were: sense, 5’-UUCUCCGAACGUGUCACGUTT-3’, and antisense, 5’-ACGUGACACGUUCGGAGAATT-3’. About 50% confluent monolayers were washed and incubated in serum- and antibiotic-free medium. All siRNA transfections were performed using Lipofectamine 2000 according to the manufacturer’s protocol.

### RNA-seq

RNAiso Plus was used for extracting total RNA from cultured cells. The RNA quality was measured using Agilent 4200 TapeStation. Then, RNA quantity was measured using Qubit 2.0 Fluorometer. RNA samples were sent to Genminix Informatic Ltd. (Shanghai, China) for mRNA sequencing on Illumina Hiseq platform (Illumina, San Diego, California, USA) with 6 Gbps. Gene-ontology functional analysis was used to categorize the functions of differentially expressed target genes.

### *H. pylori* infection of mice

Female wild-type (WT) C57BL/6 mice were purchased from the Experimental Animal Centre of the Third Military Medical University. For infecting mice, *H. pylori* were amplified in Brucella broth with 5% fetal bovine serum with gentle shaking at 37 °C under microaerobic conditions. Twenty hour later, live bacteria were collected and adjusted to 10^9^ colony forming units (CFU)/mL. Mice were fasted overnight and orogastrically inoculated twice at a 1-day interval with 5 × 10^8^ CFU bacteria. Five mice per group per time point. All breeding and experiments were reviewed and approved by the Animal Ethical and Experimental Committee of Third Military Medical University.

### Immunohistochemistry

Mouse and human gastric tissue sections were deparaffinized, rehydrated and boiled in citrate buffer. Sections were incubated with primary anti-ETS1 Ab overnight at 4 °C. The primary Ab was detected using a biotinylated secondary Ab followed by incubation with horseradish peroxidase-conjugated avidin to form avidin-biotin complexes according to the manufacturer’s protocol. Slides were then stained with DAB, counterstained with haematoxylin and photomicrographs captured using a bright field microscope (Nikon Eclipse 80i; Nikon, Tokyo, Japan).

### Antibodies and other reagents

Details are available in the Supplementary Table 4.

### Ethical statement, patients, and specimens

The gastric biopsy specimens were gathered from 73 patients (53 *H. pylori*-infected patients and 20 uninfected volunteers) who underwent upper esophagogastroduodenoscopy for dyspeptic symptoms at Xinqiao Hospital. The grade of gastritis (mild, moderate and severe) was based on the density of infiltrating mononuclear and polymorphonuclear cells according to the updated Sydney System^36^. The histopathological analysis was carried out by two experienced histopathologists. All experimental procedures were approved by the Third Military Medical University’s human ethics committee and written informed consent obtained from each patient. The clinical characteristics of all patients were described in Supplementary Table 5.

### Statistical analysis

Quantitative data are presented as the mean ± SEM. Generally, Student *t*-test was used to analyze the differences between two groups; however, when the variances differed, the Mann–Whitney *U* test was used. All data were analyzed using the GraphPad Prism software version 5.0. For RNA-seq analysis, the differentially expressed genes were selected as having more than two-fold difference between their geometrical mean expression in the compared groups and a statistically significant *P*-value (<0.05) by analysis of DEseq2. Statistical significance was defined when *P* < 0.05.

## Supplementary information

Supplementary Figure 1

Supplementary Figure 2

Supplementary Figure Legends

Supplementary Table 1

Supplementary Table 2

Supplementary Table 3

Supplementary Table 4

Supplementary Table 5
