# Supplementary material for: Expression of ETS1 in gastric epithelial cells positively regulate inflammatory response in Helicobacter pylori-associated gastritis
Source: Cell Death Dis. 2020 Jul 1;11(7):498. doi: 10.1038/s41419-020-2705-8 (PMC7329872; doi:10.1038/s41419-020-2705-8)
Supplement: Supplementary file 3 — Supplementary Figure Legends [file 41419_2020_2705_MOESM3_ESM.doc]

**SUPPLEMENTARY FIGURES**

**Supplementary Figure 1**

**a** AGS cells were pre-treated with BAY 11-7082 and then stimulated with *H. pylori* 11637 (MOI=100) for 3 h. The p65 and p-p65 protein levels were analyzed by Western blot. **b** AGS cells were pre-treated with BAY 11-7082 and then stimulated with *H. pylori* 26695 (MOI=100) for 3 h. The p65 and p-p65 protein levels were analyzed by Western blot.

**Supplementary Figure 2**

**a** IL-1β mRNA in gastric mucosa of *H pylori* 11637-infected and uninfected mice at 12 week p.i. were analyzed by real-time PCR (n=5). **b** TNFα mRNA in gastric mucosa of *H pylori* 11637-infected and uninfected mice at 12 week p.i. were analyzed by real-time PCR (n=5).
