# Supplementary material for: Expression of ETS1 in gastric epithelial cells positively regulate inflammatory response in Helicobacter pylori-associated gastritis
Source: Cell Death Dis. 2020 Jul 1;11(7):498. doi: 10.1038/s41419-020-2705-8 (PMC7329872; doi:10.1038/s41419-020-2705-8)
Supplement: Supplementary file 4 — Supplementary Table 1 [file 41419_2020_2705_MOESM4_ESM.doc]

**Supplementary Table 1.** ETS family genes obtained from HUGO Gene Nomenclature Committee (HGNC) database.

| HGNC ID (gene) | Approved symbol | Approved name | Previous symbols | Synonyms | Chromosome |
| --- | --- | --- | --- | --- | --- |
| HGNC:3246 | EHF | ETS homologous factor |  | ESE3,ESEJ | 11p13 |
| HGNC:3316 | ELF1 | E74 like ETS transcription factor 1 |  |  | 13q14.11 |
| HGNC:3317 | ELF2 | E74 like ETS transcription factor 2 |  | EU32,NERF,NERF-2,NERF-1A,NERF-1B | 4q31.1 |
| HGNC:3318 | ELF3 | E74 like ETS transcription factor 3 | ESX | EPR-1,ESE-1,ERT | 1q32.1 |
| HGNC:3319 | ELF4 | E74 like ETS transcription factor 4 |  | MEF,ELFR | Xq26.1 |
| HGNC:3320 | ELF5 | E74 like ETS transcription factor 5 |  |  | 11p13 |
| HGNC:3321 | ELK1 | ETS transcription factor ELK1 |  |  | Xp11.23 |
| HGNC:3325 | ELK3 | ETS transcription factor ELK3 |  | ERP,NET,SAP2 | 12q23.1 |
| HGNC:3326 | ELK4 | ETS transcription factor ELK4 |  | SAP1 | 1q32.1 |
| HGNC:3444 | ERF | ETS2 repressor factor |  | PE-2,PE2 | 19q13.2 |
| HGNC:3446 | ERG | ETS transcription factor ERG |  | erg-3,p55 | 21q22.2 |
| HGNC:3488 | ETS1 | ETS proto-oncogene 1, transcription factor | EWSR2 | FLJ10768,ETS-1 | 11q24.3 |
| HGNC:3489 | ETS2 | ETS proto-oncogene 2, transcription factor |  |  | 21q22.2 |
| HGNC:3490 | ETV1 | ETS variant transcription factor 1 |  | ER81 | 7p21.2 |
| HGNC:3491 | ETV2 | ETS variant transcription factor 2 |  | ER71 | 19q13.12 |
| HGNC:3492 | ETV3 | ETS variant transcription factor 3 |  | PE-1 | 1q23.1 |
| HGNC:33834 | ETV3L | ETS variant transcription factor 3 like |  | FLJ16478 | 1q23.1 |
| HGNC:3493 | ETV4 | ETS variant transcription factor 4 |  | E1A-F,E1AF,PEA3 | 17q21.31 |
| HGNC:3494 | ETV5 | ETS variant transcription factor 5 |  | ERM | 3q27.2 |
| HGNC:3495 | ETV6 | ETS variant transcription factor 6 |  | TEL | 12p13.2 |
| HGNC:18160 | ETV7 | ETS variant transcription factor 7 |  | TEL2,TEL-2 | 6p21.31 |
| HGNC:18562 | FEV | FEV transcription factor, ETS family member |  | Pet-1 | 2q35 |
| HGNC:3749 | FLI1 | Fli-1 proto-oncogene, ETS transcription factor |  | SIC-1,EWSR2 | 11q24.3 |
| HGNC:4071 | GABPA | GA binding protein transcription factor subunit alpha |  | E4TF1A,NFT2,NRF2,E4TF1-60,NRF2A | 21q21.3 |
| HGNC:17257 | SPDEF | SAM pointed domain containing ETS transcription factor |  | PDEF,bA375E1.3 | 6p21.31 |
| HGNC:11242 | SPIB | Spi-B transcription factor |  | SPI-B | 19q13.33 |
| HGNC:29549 | SPIC | Spi-C transcription factor |  | MGC40611,SPI-C | 12q23.2 |
| HGNC:11241 | SPI1 | Spi-1 proto-oncogene |  | PU.1,SPI-A,OF,SFPI1,SPI-1 | 11p11.2 |
