# Supplementary material for: Expression of ETS1 in gastric epithelial cells positively regulate inflammatory response in Helicobacter pylori-associated gastritis
Source: Cell Death Dis. 2020 Jul 1;11(7):498. doi: 10.1038/s41419-020-2705-8 (PMC7329872; doi:10.1038/s41419-020-2705-8)
Supplement: Supplementary file 5 — Supplementary Table 2 [file 41419_2020_2705_MOESM5_ESM.doc]

**Supplementary Table 2.** Promoter sequence used for analysis

| Promoter sequence (-2000 to 0) |
| --- |
| **Human ETS1 promoter sequence (5’-3’)**  GGATGATTGACTATCTGAGTTGTCACTATGGAAGAATTAGAAAGAATAATTTTCCTCATT  GCTAGAGTGGAAAGTGTTTACAGTTTAATCATGGTGGGCTTTTTTAGTATCTAATTTGGG  TGGATGTTAGCAGCAGTGTTTAGGCTAGTCTGGGGAAGAAAGTTGAGGAGCAAACTGGGG  AAGAGAGAAAGGAGTTGCTCTGAGGAATCTGCATTTGAAAATAGAGAAGTACTCACACTT  GGATTAAAAAATGCCAACATTTTCTCAATACAACATCAACAAAACAAAATTCCCCAAAGC  AACTCTAGATTTGTTCACTTCTTCTCCTGATGAATTCATAGTGAGTAAGGCAGAATTGAT  TCAAGAACCTTTTTTTTTTTGTGGTTCATTTGGACGTGTAAATGTACAATTCATTCATCA  GCTGTTTCTCTAGGCTTTTCCCAGGACCAGAAGGGTCTGTTCTTGTCATCTGGAGGCCAG  CATTGTTTTCTCATACCTTGTGGAAACACAGCTGGATTGCAACAGTCTTTGTTCTTTCAA  AAATTAGGACTATCACATACAAATATTTTCCTCTGGTCACTAAGGAGGGATTTAATCTGG  AGTTTAGACCAGGAGCCACAGATTTTTCTGTTCCTGGCTTCCTCCCCGATTTGCTGTGGG  AAATCTGCAAATTACTAAGGCTTCAAGGGCACAAAACTGGGATTAGATGAAGGTAAGCCT  GTGTGTCAAAACTTACCAGAGGCAGAGGTTACAGTTACAGTCAAATGCTGGGAGTTCAAA  TGTGCTTAGAAATCAGGAAACATTAAAAATAGCTTTCTCTGAGATTGCTCAGGAAGATTT  ATGTTTAATCTTTCTGTTAATCAAAGATGGAGCTCAGTGCCTGGTTCTAGCTATGTATTT  AGATATATACATGTACTGGTTGATTTGTTAATTAGGACAAACTATCTCTATTACAACCAA  TGTATTAGTGGCAAACCATGAATTATTCGGGTTCATTTTGTTTTATCTCTAGTGTTGGGG  GTTCCCAAATGGAACAGAAACTGTGCATTGTGATTCTGTCATGGCTGCCAATGATGTACT  CCAGAAATTCAAAACCACAGAGCCTCTGCATCACTTTTTTGGTGGCGGTCGTGGGAGGGT  TGTTAGTTTTATCTAAAAGAGGAGAGTCTGTGAAAGGAAAGACAAAGCTTTGGCCAATGG  TGACATAATTAGAGGAACAGAATAATTAGAAATAAATCATAACTTCCCCTTCAACCCAAC  CACTGCTGGAGGTAAATTGGAAGCTTACGGAAGCTCATGGCTGGGTTCTAGTTTGGGGTC  CCGTTCTGGACTCCCCTGCCAACGCAGGAGCATTACATGGGTGGGCTCTGAAAAACTGAA  GCCTCCTTCAGAAACACACGCCCTGACTCAAGATCCGGCTGGAGTCCAATACTCCTAAAG  CCCTTTGAGGACACGGGCTCACGAATCCCCTGCGCCTGCCTGCACGCTCGCCTTCATCCA  CATGCCTCACGTCCTGTGTGTCAGTCTTTGTGAATGAATGATGTACACGCACTTGGAAAA  CTATGCTGCTACTGGGAGGGGGCGAGAGCGGGTGACCAAGCCCTCAAGAATGCGTGGAGA  ATCAGACGGACTTTCCCGAAACGGTGGAGGCGGCCTGTGCACCCAGCCTGCACACCCGCT  CCCGGCCCTTCCCGCCCCTGCCTGGGCTCCGAGGCCCGGGGCTCCACGCACTGCTCCTCC  GCGGTCGCGGCCCGGCCCCGCGCTCGCCAGCCCTTCCTTTCGCTTTGGGCCGCGGGCGGA  GATTGGCCGCGGTGCTCGGCCCCGCCCCCGGCTCCCCGCCCTGCCCCGACGCCCCGCCCC  TCGCTCCCCCAAGCGGACATCGCCCGGCCCAGGGCGCGCGGACCCAGCGGCCGAGACGCG  GCGCCGCGAGAAACGCCCTAAAGAGGAGGGGAGAGCGCGGGAGGGCGAGAGGGAGGGAGA  GCGGCCGGGAGATCGAGAGCG |
