# Supplementary material for: Expression of ETS1 in gastric epithelial cells positively regulate inflammatory response in Helicobacter pylori-associated gastritis
Source: Cell Death Dis. 2020 Jul 1;11(7):498. doi: 10.1038/s41419-020-2705-8 (PMC7329872; doi:10.1038/s41419-020-2705-8)
Supplement: Supplementary file 6 — Supplementary Table 3 [file 41419_2020_2705_MOESM6_ESM.doc]

**Supplementary Table 3.** Primer sequences used for real-time PCR analysis

| Gene |  | Primer sequence 5′→3′ |
| --- | --- | --- |
| Human *GAPDH*  Human *ETS1*  Mouse *β-actin*  Mouse *Ets1*  Mouse *Il1β*  Mouse *Tnfa* | forward  reverse  forward  reverse  forward  reverse  forward  reverse  forward  reverse  forward  reverse | ACCCAGAAGACTGTGGATGG  CAGTGAGCTTCCCGTTCAG  AGCTTCGACTCAGAGGACTATCCG  GGCAGCAGCAGGAATGACAGG  AGTGTGACGTTGACATCCGT  GCAGCTCAGTAACAGTCCGC  AGAAGTTCTGTATGAGTGGAGC  CCTCTTTCTGCAGGATCTCTAG  TCGCAGCAGCACATCAACAAGAG  AGGTCCACGGGAAAGACACAGG  ATGTCTCAGCCTCTTCTCATTC  GCTTGTCACTCGAATTTTGAGA |
