# Supplementary material for: Expression of ETS1 in gastric epithelial cells positively regulate inflammatory response in Helicobacter pylori-associated gastritis
Source: Cell Death Dis. 2020 Jul 1;11(7):498. doi: 10.1038/s41419-020-2705-8 (PMC7329872; doi:10.1038/s41419-020-2705-8)
Supplement: Supplementary file 7 — Supplementary Table 4 [file 41419_2020_2705_MOESM7_ESM.doc]

**Supplementary Table 4**. Antibodies and other reagents

| **Antibodies and reagents** | **Manufacturers** |
| --- | --- |
| Antibodies for western blot  Rabbit anti-human/mouse GAPDH (#5740, 1:1000)  Rabbit anti-human/mouse ETS1 (#14069, 1:1000)  Rabbit anti-human p65 (#8242, 1:1000)  Rabbit anti-human p-p65 (#3033, 1:1000)  HRP-conjugated goat anti-rabbit IgG Abs (#ZB-2301, 1:10000,) | Cell Signaling Technology, USA  Cell Signaling Technology, USA  Cell Signaling Technology, USA  Cell Signaling Technology, USA  Zhong Shan Gold Bridge Biotechnology, China |
| Antibodies for Immunohistochemistry  Rabbit anti-human/mouse ETS1 (#ab26096, 1:100) | Abcam, USA |
| Antibodies for ChIP assay  Rabbit anti-human/mouse p65 (#8242, 1:100)  Rabbit IgG isotype control (#ab172730, 1:100) | Cell Signaling Technology, USA  Abcam, USA |
| Recombinant human cytokines  Recombinant human IFN-γ  Recombinant human IL-17A  Recombinant human IL-22  Recombinant human IL-6  Recombinant human IL-12  Recombinant human IL-23  Recombinant human IL-1β  Recombinant human TNFα | PeproTech, USA  PeproTech, USA  PeproTech, USA  PeproTech, USA  PeproTech, USA  PeproTech, USA  PeproTech, USA  PeproTech, USA |
| Human CD326 microbeads  Collagenase IV  DNaseI  Fetal bovine serum (FBS)  RPMI-1640  DMEM/F12  Penicillin/Streptomycin  0.4-μm pore size Transwells  BAY 11-7082  DMSO  RNAiso Plus  PrimeScriptTM RT reagent Kit  SYBO Green Real-time PCR Master Mix  RIPA Lysis and Extraction Buffer  Dual-Luciferase Reporter assay  Lipofectamine™ 2000 Transfection Reagent  EZ-Magna ChIP™ A/G Chromatin Immunoprecipitation Kit  Tissue Protein Extraction Reagent  DAB    Biotin-Streptavidin HRP Detection Kits    Haematoxylin | MilteniyBiotec, Germany  Gibco, USA  Sigma-Aldrich, USA  PAN Biotech, Germany  Hyclone, USA  Hyclone, USA  Beyotime Biotechnology, China  Corning, USA  Calbiochem, USA  Sigma-Aldrich, USA  TaKaRa, Japan  TaKaRa, Japan  Toyobo, Japan  Thermo, USA  Promega, USA  Invitrogen, USA  Merck Millipore, Germany  Pierce, USA  Zhong Shan Gold Bridge Biotechnology, China  Zhong Shan Gold Bridge Biotechnology, China  Sangon Biotech, China |
