# Supplementary material for: Expression of ETS1 in gastric epithelial cells positively regulate inflammatory response in Helicobacter pylori-associated gastritis
Source: Cell Death Dis. 2020 Jul 1;11(7):498. doi: 10.1038/s41419-020-2705-8 (PMC7329872; doi:10.1038/s41419-020-2705-8)
Supplement: Supplementary file 8 — Supplementary Table 5 [file 41419_2020_2705_MOESM8_ESM.doc]

**Supplementary Table 5.** Clinical characteristics of patients.

| Variables | Uninfected | *H. pylori-*infected |
| --- | --- | --- |
| Age (median, range)  Sex (male/female) | (40 year, 33-63 years)  7/13 | (52year, 18-65 years)  21/32 |

Exclusion criteria were: previous treatment for *H. pylori* infection, use antibiotics and/or acid secretion inhibitors during the 2 months before the study, use of anticoagulant drugs in the last week, long-term use of corticosteroids or anti-inflammatory drugs, severe concomitant cardiovascular, respiratory or endocrine diseases, gastrointestinal malignancy, clinically significant renal or hepatic disease, haematological disorders, previous gastro-oesophageal surgery, pregnancy or lactation, history of allergy to any of the drug used in the study, alcohol abuse, drug addiction, and severe neurological or psychiatric disorders.
